# Haemorrhagic Pericardial Effusion Following Intrapleural Fibrinolysis Therapy With Alteplase/Dornase Alfa for Complicated Parapneumonic Effusion: A Case Report

**DOI:** 10.7759/cureus.87186

**Published:** 2025-07-02

**Authors:** Jesheen Mann, Leshanth Uthayanan, Vikas Gulia

**Affiliations:** 1 Internal Medicine, George Eliot Hospital NHS Trust, Nuneaton, GBR; 2 Anaesthesia, George Eliot Hospital NHS Trust, Nuneaton, GBR

**Keywords:** cardiac tamponade, complicated parapneumonic effusion, intrapleural fibrinolysis, pericardial effusion, pericardial haemorrhage

## Abstract

Standard treatment for complicated parapneumonic effusion is antibiotic therapy and drainage of the infected pleural fluid. Complicated effusions refractory to standard treatment require further interventions such as intrapleural fibrinolysis. This is effective for patients with loculated effusions as local fibrinolytics administered via a chest drain can break down the septations. Common complications include intrapleural bleeding, hypersensitivity, pain and discomfort with fibrinolytics, but no studies, to our knowledge, have reported pericardial haemorrhage. In this case report, we describe a 75-year-old female who presented with dyspnoea, mucopurulent cough and intermittent fever. She was diagnosed with community-acquired pneumonia. A chest radiograph revealed pleural effusion suggestive of parapneumonic effusion. Further computed tomography (CT) imaging showed a complicated parapneumonic effusion with loculations that were drained by an ultrasound-guided chest drain. As there was inadequate drainage, local fibrinolytics were administered. On the third administration of alteplase and dornase alfa, the patient deteriorated with a drop in haemoglobin and presented with muffled heart sounds, jugular venous distension, and low-normal systolic blood pressure (Beck's triad). A clinical suspicion of pericardial effusion was made and confirmed by echocardiography. CT imaging revealed a high-density effusion as measured with the Hounsfield Unit, supporting the likelihood of a haemorrhagic pericardial effusion. Subsequently, the patient was stabilised with conservative management, close observation, and input from the intensive treatment unit team. In conclusion, we report pericardial haemorrhage as a rare complication of local fibrinolytic therapy that can be managed conservatively.

## Introduction

Pleural infection represents a spectrum of conditions with a reported 30-day mortality of up to 10.5% [1,2]. Parapneumonic effusions are seen in 20-40% of patients treated for pneumonia in the hospital [3]. Simple parapneumonic effusion is pneumonia with pleural effusion, and becomes complicated when there is an infection of the pleural space. The diagnosis is usually made through assessing clinical parameters, infection markers and pleural fluid sampling. The most accurate biomarker is when the pleural fluid pH is <7.2. They are also classified as complicated if they require chest tube drainage for resolution and/or have positive pleural fluid cultures [4]. Standard treatment protocols for complicated parapneumonic effusion are broad-spectrum antibiotic therapy and drainage of the infected pleural fluid using a chest tube. Approximately one-third of patients require surgical intervention when chest tube drainage and antibiotic treatment prove ineffective [5]. Parapneumonic effusions occur in approximately 60% of patients who are hospitalized with community-acquired pneumonia, although only a subset progress to complicated effusions requiring drainage or intrapleural fibrinolysis. There have been studies to support the role of intrapleural fibrinolysis when drainage and infection clearance were sub-optimal with standard treatment protocols [6-11].

Studies have discussed common complications such as intrapleural bleeding, hypersensitivity, pain and discomfort following intrapleural fibrinolysis [5,11]. However, none to the best of our knowledge have reported pericardial haemorrhage. We describe a case of complicated parapneumonic effusion that was managed with intrapleural alteplase and dornase alfa (deoxyribonuclease, DNAse), given via the chest drain, to break down local septations and allow effective chest-tube drainage. There was an unexpected complication in the development of haemorrhagic pericardial effusion that was managed conservatively and had a favourable outcome. We highlight that pericardial haemorrhage is a rare complication of intrapleural fibrinolysis that can be managed conservatively with serial monitoring.

## Case presentation

A 75-year-old female presented with a seven-day history of dyspnoea, mucopurulent productive cough, and intermittent fever. She had no significant travel history and no notable past medical conditions, except for a history of smoking, which she reports to have ceased years ago. She also had no notable occupational history. Her Rockwood Frailty Score was 2, indicating moderate frailty.

On admission, the patient was significantly hypoxic, with an oxygen saturation of 85%, and tachycardic (heart rate 120 beats per minute). She required high-flow oxygen at 10 litres via a face mask to meet oxygen saturations >94%. Physical examination revealed signs of respiratory distress and mild peripheral oedema. Blood investigations showed an elevated white cell count with neutrophilia, indicating an ongoing bacterial infection (Table 1). Inflammatory markers, including white blood cell (WBC), neutrophils and C-reactive protein (CRP), were significantly raised, while her haemoglobin and albumin were within normal limits, although the latter was at the lower end, likely due to ongoing inflammatory processes. Baseline coagulation parameters were normal. 

**Table 1:** Blood laboratory investigation results on the dates of admission, chest drain insertion, after fibrinolysis and discharge.

Bloods	On Date of Admission	On Date of Chest-Drain Insertion	Post-Fibrinolysis	On Date of Discharge
Haemoglobin (grams/litre)	138	113	92	108
White Blood Cell (X10^9^/litre)	33.58	24.42	21.09	7.14
Neutrophils (X10^9^/litre)	32.44	21.59	17.90	4.10
Lymphocytes (X10^9^/litre)	0.47	1.15	1.20	1.28
Platelets (X10^9^/litre)	473	1203	1546	786
International Normalised Ratio	1.5			-
C-reactive protein (milligrams/litre)	346	286	215	28
Sodium (milimoles/litre)	134	136	134	138
Potassium (milimoles/litre)	3.8	4.1	4.1	4.4
Urea (milimoles/litre)	14.3	4.1	5.1	4.7
Creatinine (micromoles/litre)	158	50	41	40
Total Protein (grams/litre)	68	61	56	72
Albumin (grams/litre)	35	27	25	36
Bilirubin (micromoles/litre)	9	6	3	3
Alkaline Phosphatase (units/litre)	74	232	159	78
Alanine Transaminase (units/litre)	18	45	27	22
N-terminal pro B-type Natriuretic Peptide (picomoles/litre)	818	-	-	-

**Figure 1:**
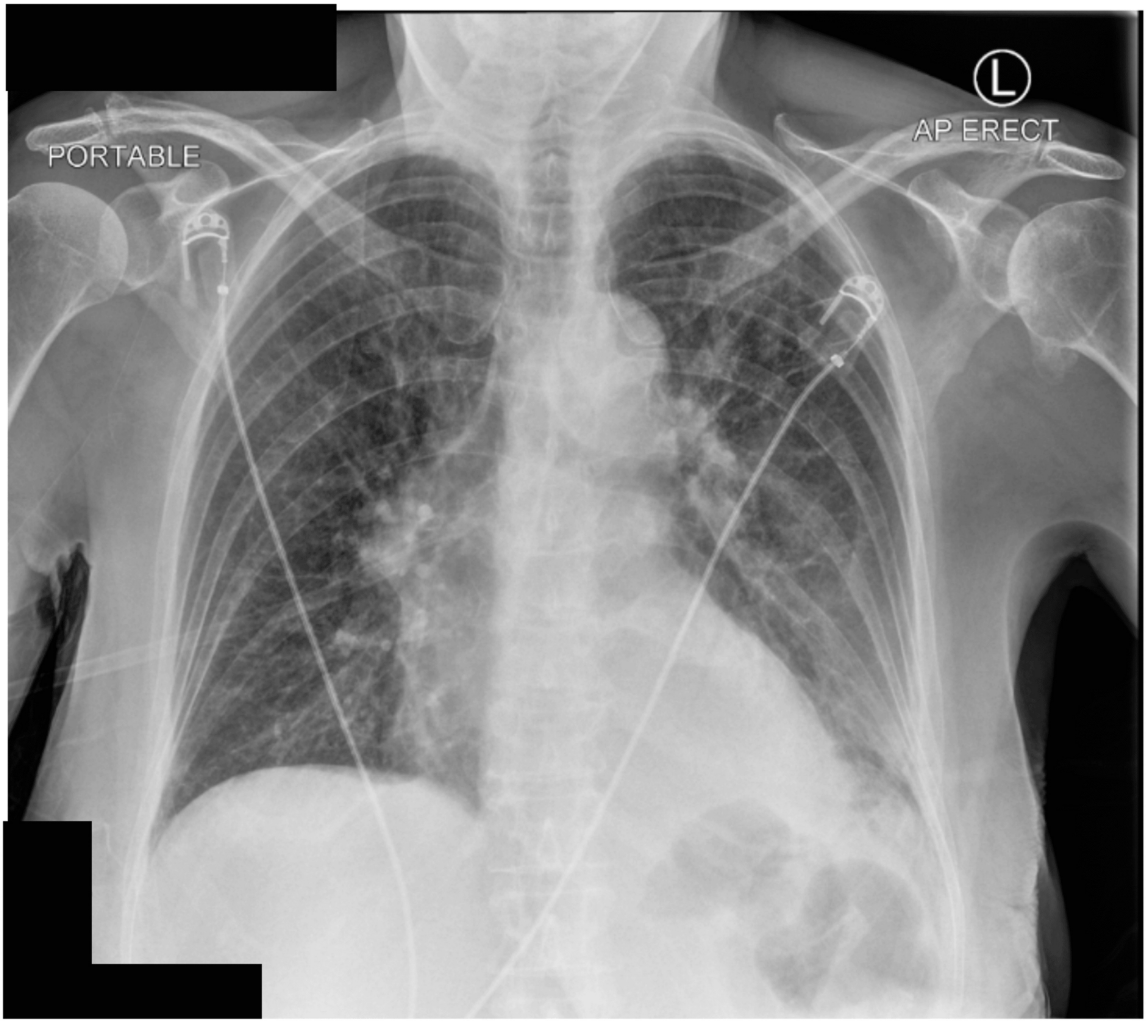
A chest radiograph demonstrating patchy opacification in the lower zone of the left lung and blunting of the costophrenic and cardiophrenic angles, suggesting pleural effusion

A chest radiograph demonstrated patchy opacification in the lower zone of the left lung with blunting of the costophrenic and cardiophrenic angles, suggesting a possible pleural effusion (Figure 1). A subsequent contrast-enhanced CT scan confirmed a moderate left-sided pleural effusion, underlying atelectasis, and ground-glass multifocal consolidations, suggesting complicated parapneumonic effusion.

Given the clinical and radiological findings, the patient was diagnosed with a complicated parapneumonic effusion. A chest drain was inserted under ultrasound guidance, but failed to drain adequately. Due to the loculated nature of the pleural effusion on ultrasound, fibrinolytic therapy was considered. Thick, viscous, mucopurulent fluid was drained with a low pH, confirming an infective aetiology. Pleural fluid analysis revealed a predominance of neutrophils; blood cultures grew *Streptococcus pneumoniae*.

The patient was started on intravenous vancomycin, empirically targeting community-acquired pneumonia. Despite adequate antibiotic therapy, the patient’s infection markers, particularly the WBC count and CRP, showed minimal improvement, and chest drain output remained inadequate. A bedside ultrasound showed loculated effusion, leading to the initiation of once-daily intrapleural fibrinolysis with alteplase and dornase alfa via the chest drain to break down the loculations and facilitate drainage.

Following the third dose of fibrinolytic therapy, the drain was predominantly haemorrhagic, and there was an acute drop in haemoglobin levels from a baseline of 138 to 92. Her condition worsened with increasing oxygen requirements (up to 15 litres) and the development of atrial fibrillation. Clinical examination revealed muffled heart sounds, jugular venous distension (JVD), and low-normal systolic blood pressure (Beck's triad), leading to a clinical suspicion of cardiac tamponade.

**Figure 2:**
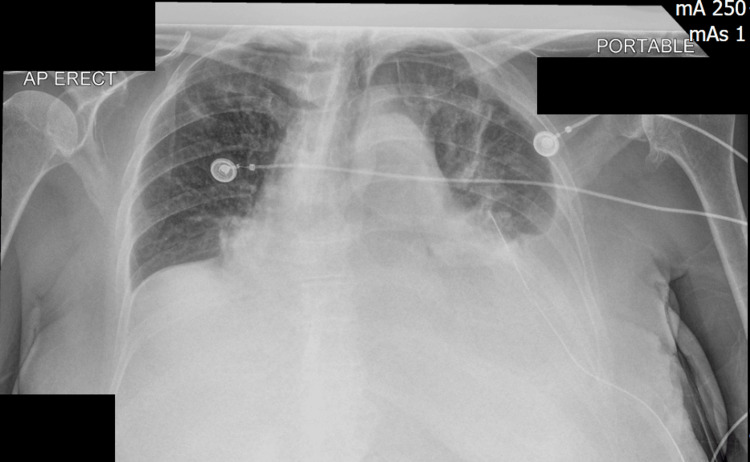
A chest radiograph showing increasing left-sided pleural effusion, new right-sided pleural effusion and increased cardiac silhouette

A repeat chest radiograph demonstrated an increase in the left pleural effusion, a new right-sided pleural effusion, and an enlargement of the cardiac silhouette (Figure 2).

**Figure 3:**
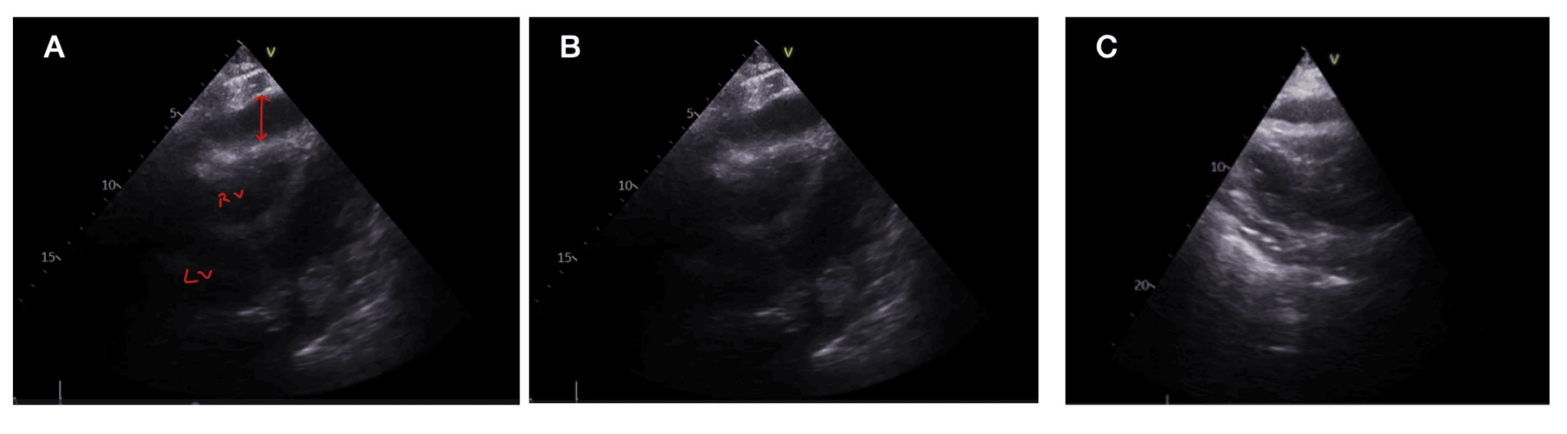
Echocardiography images suggestive of pericardial effusion A) Parasternal short-axis window. Double-sided arrow demonstrating pericardial effusion. B) Parasternal short axis. C) Parasternal long axis (PLAX) of diastolic collapse LV - left ventricle, RV - right ventricle.

This clinical presentation and elevated B-type natriuretic peptide (BNP) led to an urgent bedside echocardiogram. The echocardiogram revealed a 2.04 cm pericardial effusion, right ventricular diastolic collapse and a dilated inferior vena cava (IVC) (Figure 3). Additionally, the blood flow variation on inspiration across the mitral valve during the respiratory cycle was consistent with acute pericardial effusion. Following the echocardiographic findings, fibrinolytic therapy was discontinued, and a balanced fluid resuscitation strategy was implemented, accompanied by close monitoring with the involvement of the intensive treatment unit team. The patient received supportive treatment with tranexamic acid and albumin infusion to improve their intravascular volume and oncotic pressure. Regular arterial blood gases were done to evaluate hypoxia and respiratory acidosis.

**Figure 4:**
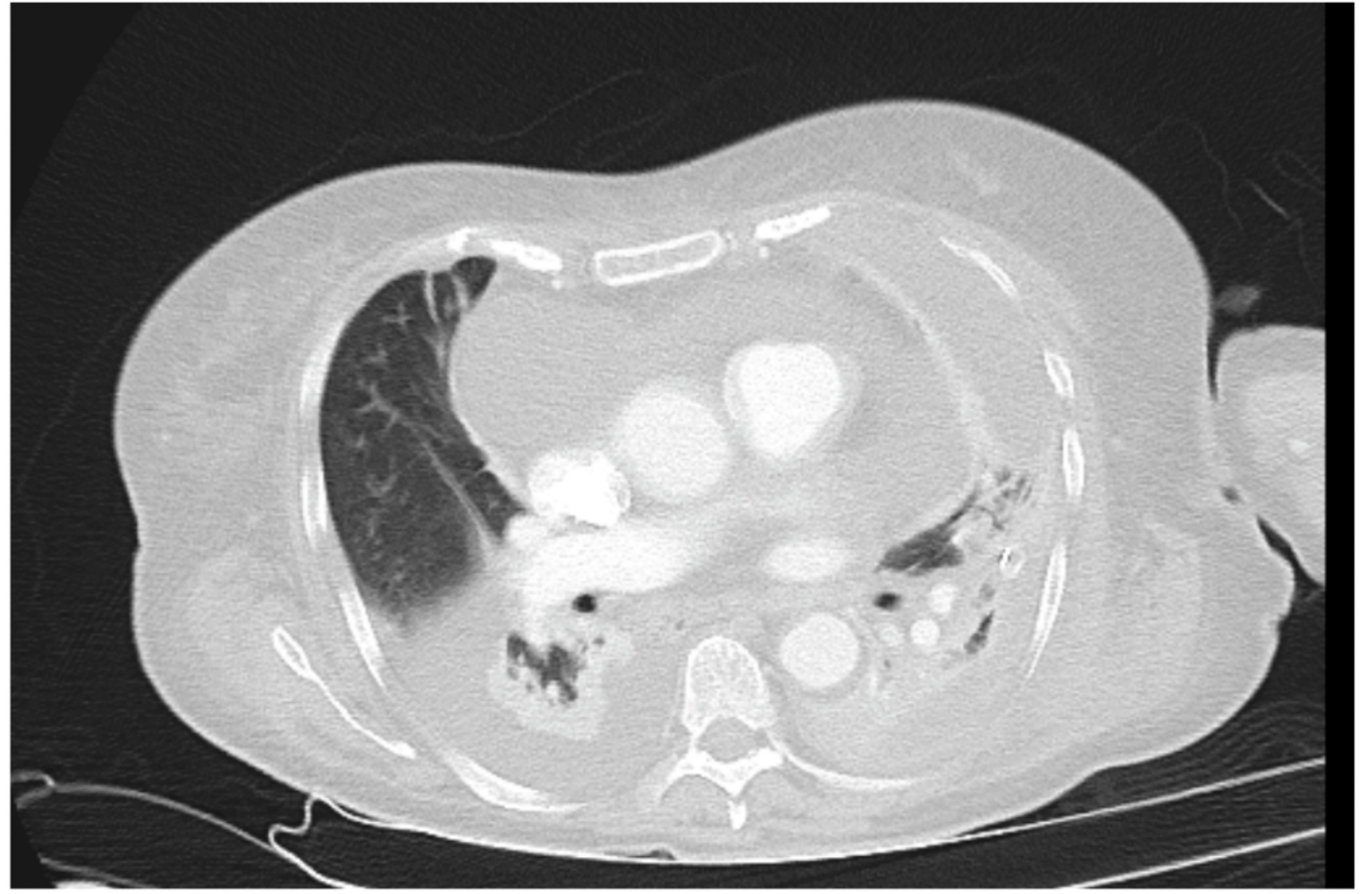
CT showing high-density loculated effusion on the right side and low-density effusion on the left side as observed by Hounsfield Units

After the patient became haemodynamically stable, CT chest revealed a persistent high-density loculated effusion on the left side, measuring 27 Hounsfield Units (HU) and a low-density right-sided effusion of 10 HU (Figure 4). Notably, the pericardial effusion demonstrated higher density (22 HU), consistent with subacute haemorrhage and possible ongoing slow bleeding.

The patient underwent daily monitoring with ultrasound, which showed gradual resolution of the pericardial effusion, and no further signs of tamponade were noted. Over the following days, the patient's clinical condition began to improve. Her oxygen requirements decreased; she maintained oxygen saturation at 92% in room air. There were no further signs of hemodynamic instability, and her blood parameters gradually normalised. Follow-up chest X-ray showed improvement of the effusion. 

## Discussion

This case highlights the complexity of managing complicated parapneumonic effusions with local intrapleural fibrinolytics. The radiological CT imaging revealed a high-density pericardial effusion. Notably, the density of the pericardial effusion closely resembled the pleural haemorrhage, where the inserted chest drain revealed visible evidence of haemorrhagic pleural fluid. This was further supported by a sudden decline in haemoglobin level compared to admission. This suggested that there was a possible subacute haemorrhage with slow ongoing bleeding, allowing the body to maintain hemodynamic stability. 

Previous research has highlighted common complications such as intrapleural bleeding, hypersensitivity, pain and discomfort [5,11]. However, a review of the literature has reported no cases of pericardial haemorrhage following intrapleural fibrinolysis with alteplase and dornase alfa.

Given the rarity of this complication, it is difficult to establish the pathophysiology from the current literature. We propose several plausible mechanisms to explain this pericardial haemorrhage. One possible mechanism is the cross-membrane spread of fibrinolytic agents. There have been several reports of extrapleural bleeding following intrapleural fibrinolysis, including two episodes of gastrointestinal bleeding in the MIST2 trial [5]. The authors of a case report suggested systemic absorption of tissue plasminogen activator had caused severe gastrointestinal bleeding following intrapleural fibrinolysis [12]. Since the pleura and pericardium are anatomically close, the intrapleural administration of alteplase and dornase alfa may lead to systemic absorption or diffusion into the pericardial space, causing haemorrhagic complications. Another plausible explanation is that secondary to parapneumonic effusion, its associated inflammation may extend to the pericardium, rendering its vessels fragile and prone to rupture on fibrinolysis. In patients who underwent intrapericardial fibrinolysis for pericardial effusion, the most frequent short-term complication was haemorrhage, as a result likely due to pericardial-induced inflammation and fibrinolysis [13].

Thirdly, the presence of Streptococcus infection in the bloodstream raises the possibility of early septic coagulopathy, which may have further increased the haemorrhagic risk associated with fibrinolytic therapy.

A study assessing bleeding complications in patients with intrapleural fibrinolysis reported systemic anticoagulation, high RAPID (renal, age, purulence, infection source, and dietary factors) score, high serum urea level, and platelets <100 x 10^9^/ L to be associated with an increased bleeding risk [14]. Interestingly, our patient did not meet any of these criteria and had no deranged renal or hepatic function, thus highlighting the rarity of this presentation. 

Pericardial haemorrhage can lead to tamponade [15]. Approximately 21% of cases of cardiac tamponade are iatrogenic [16]. Medical causes can be secondary to fibrinolytic therapy, anticoagulation, or anti-cancer therapy. Surgical causes of cardiac tamponade can be due to catheter-based procedures, such as catheter ablation, pacemaker implantation, and central venous catheter placement [16].

Cardiac tamponade develops due to fluid accumulation in the pericardial space, leading to restricted diastolic filling of the heart. The rate at which this fluid accumulates determines the severity of its impact. When the fluid build-up is gradual, the pericardium has time to adapt and stretch, thus allowing for a greater volume to accumulate. When this process happens over a shorter duration, even a small volume can surpass the pericardium’s adaptive capacity and lead to hemodynamic instability [16]. As seen in this case, the patient experienced an increasing oxygen requirement and a drop in systolic blood pressure, indicating progression towards cardiac tamponade. However, prompt action successfully prevented further deterioration.

It is important to recognise that the incidence and outcomes of parapneumonic effusions can vary significantly across different populations. Individuals from underprivileged, low-resource settings or alcohol-dependent backgrounds face increased risk due to delayed healthcare access, malnutrition, poor dental hygiene and chronic co-morbidities [17,18]. These social determinants may inadvertently contribute to the development of parapneumonic effusion and influence the severity of other complications such as pericardial effusion and cardiac tamponade [18,19]. 

The usual management for cardiac tamponade is ultrasound-guided pericardiocentesis [16,18-20]. In our case, we adopted a conservative approach, with balanced fluid resuscitation and close monitoring, since the patient tolerated the effusion and was hemodynamically stable [17]. Prompt recognition and the decision to discontinue fibrinolytic therapy helped prevent the progression of haemorrhagic pericardial effusion into frank cardiac tamponade. This is supported by the improvement seen from follow-up imaging. 

## Conclusions

In conclusion, bleeding complications should be considered for patients receiving local fibrinolytic therapy for complicated parapneumonic effusions refractory to standard treatment. We report pericardial haemorrhage, a rare complication not previously reported in patients acutely deteriorating following local fibrinolysis. Early recognition, cessation of intrapleural fibrinolysis and conservative management through close monitoring and haemodynamic support resulted in favourable outcomes. 
